# Still unsafe: what’s holding us back on online safety for women

**DOI:** 10.1007/s43681-026-01097-0

**Published:** 2026-04-10

**Authors:** Ángel Pavón Pérez, Tracie Farrell, Christine De Kock, Olga Jurasz, Debora Nozza, Miriam Fernandez

**Affiliations:** 1 Centre for Protecting Women Online, The Open University, Milton Keynes, UK; 2 Knowledge Media Institute, The Open University, Milton Keynes, UK; 3 University of Melbourne, Melbourne, Australia; 4 Bocconi University, Milan, Italy

**Keywords:** Online violence against women, Online safety, Algorithmic harm, Platform accountability, Misogyny online, TFGBV

## Abstract

Online Violence Against Women (OVAW) is a persistent and evolving challenge, encompassing AI-generated sexual content, cyberstalking, sextortion, and online harassment, among other emerging forms of abuse. Despite growing scholarly and policy attention, interventions remain limited in effectiveness. This article presents findings from the *Towards a Safer Web for Women (TSWW'25)* workshop, held at the ACM Web Conference, which convened researchers and practitioners from computer science, law, social sciences, and civil society to examine the systemic drivers of OVAW. Through participatory methods and thematic analysis, we identify two interrelated challenges: (1) systemic failures in platform governance and public regulation that undermine accountability, and (2) socio-technical dynamics that normalise misogyny and discourage bystander intervention. Our analysis highlights how profit-driven platform incentives, algorithmic optimisation, early socialisation into harmful norms, and the persistent shifting of responsibility onto victims interact to sustain OVAW. The paper articulates a set of recommendations that emerged through convergence across disciplinary perspectives. These recommendations are significant because they reflect shared priorities across fields that typically approach the problem from divergent epistemic standpoints. We argue that such cross-disciplinary consensus identifies actionable intervention points, including: protection-by-design approaches, early educational and social interventions, and sustained cross-disciplinary and cross-sector collaboration. Addressing OVAW requires an ethical re-evaluation of algorithmic governance that moves beyond isolated technical, legal, or cultural fixes toward a coordinated socio-technical framework.

## Introduction

Online Violence Against Women^1^ (OVAW)^2^ has reached critical levels worldwide, constituting a pressing societal challenge. Global data illustrates the pervasive nature of these harms, though prevalence varies by region and study design. In the Arab States, surveys of 11,500 internet users found that 60% of women have experienced online violence. Similarly, research involving over 12,000 women in Eastern Europe and Central Asia reported that 50% of women over 18 had experienced technology-facilitated abuse.^3^ In Sub-Saharan Africa, mixed-methods research including face-to-face interviews with over 3300 women indicates a 28% prevalence rate, while surveys of 4000 women across Europe and the United States report a rate of 23% [98].

The forms this violence takes are increasingly mediated by, and entangled with, advanced technologies, including AI-based systems.^4^ These online harms range from the misuse of technologies by perpetrators, such as parental control applications that enable coercive control through real-time location tracking and surveillance, to technologies that facilitate abuse, such as the algorithmic amplification of misogynistic content [81], or technologies that reinforce existing inequalities due to AI biases (e.g., lending AI-based systems reducing financial opportunities for women [74]). More recently, generative AI systems have been weaponised to intensify both the scale and severity of abuse (e.g. Grok is estimated to have produced more than 3 million sexual deepfakes [20]). Since the emergence of accessible generative AI tools, the prevalence of deepfake-related abuse has increased by over 550% [48], with non-consensual sexual imagery now accounting for 98% of all AI-generated video content online [22]. This technological shift illustrates how AI can automate, personalise, and scale harassment in ways that were previously difficult to achieve. In a landmark case, customisable AI chatbots were used to impersonate a victim by leveraging her private professional and personal data to lure strangers to her physical residence [61]. Women in public-facing roles (e.g. journalists, activists, and human rights defenders) are particularly exposed to these emerging forms of abuse: 70% report experiencing online violence, with nearly a quarter (24%) involving AI-amplified threats such as manipulated content or synthetic histories [77].

These dynamics are increasingly blurring the boundary between digital and physical harm. Evidence suggests that online attacks are progressively spilling over into offline contexts. A recent survey found that the proportion of women journalists who reported online abuse escalating into offline harassment, threats, or physical assault more than doubled in five years, rising from 20% in 2020 to 42% in 2025 [77]. These figures underscore a global crisis that, while manifesting differently across socio-technical contexts, consistently targets women.

As such, OVAW represents an important challenge: it is simultaneously technical and social, global yet deeply contextual, and shaped by the interaction between algorithmic systems, institutional governance, and human behaviour. Harmful content like hate speech and deepfakes can rapidly spread across borders, often outpacing the capacity of individual nations to regulate it effectively [23]. Linguistic diversity [38], cultural norms [95], and differing legal frameworks [35, 96, 99] pose significant challenges for the design and deployment of effective solutions. Systems trained in one cultural or linguistic context may fail to recognise harm in another, resulting in uneven protection and new forms of exclusion [89]. Despite increasing regulatory activity, there remains limited empirical understanding of how these global, contextual, and institutional factors interact to shape both the persistence of harm and the effectiveness of proposed interventions [6].

Effective responses, therefore, require interdisciplinary and cross-sectoral collaboration. Legal and regulatory frameworks, such as the UK’s Online Safety Act [96], the proposed UK legislation on deepfakes [97], and emerging European regulations including the Digital Services Act and the AI Act [35, 99], play a crucial role in establishing accountability. At the same time, technological systems can support compliance by enabling large-scale monitoring and analysis of harmful content. Policing and reporting infrastructures must also be adequately resourced to translate legal protections into meaningful remedies for victims. Beyond these institutional mechanisms, insights from the social and behavioural sciences are essential for understanding why online violence persists, how harmful norms are reproduced, and how allies and bystanders might be mobilised to intervene. Together, these perspectives highlight the need for socio-technical approaches that integrate ethical AI development with societal values, governance structures, and lived experience.

Scholars across AI ethics, feminist data studies, and legal scholarship have long argued that digital harms must be understood as socio-technical phenomena rather than purely technical failures. Feminist analyses of data and AI highlight the importance of examining power relations and centring lived experience in technological design and evaluation [32, 54], while legal scholarship argues that online harassment emerges from the interaction between platform architectures, governance gaps, and gendered social dynamics [25]. Related work in critical AI and design research further advocates participatory and justice-oriented approaches that integrate community perspectives, institutional accountability, and ethical system development [11, 16].

Building on prior work that frames digital harms as socio-technical phenomena, our study seeks to capture how these challenges are perceived and addressed across disciplinary and institutional contexts in practice. In response to these gaps, we organised the *Towards a Safer Web for Women (TSWW'25)* workshop at the ACM Web Conference, a leading international conference focused on the web, digital technologies, and their societal impacts, with over 1000 attendees from multiple disciplines and sectors. The workshop was explicitly designed as an interdisciplinary forum bringing together researchers and practitioners from diverse geographical and disciplinary backgrounds to examine OVAW. Rather than proposing a single technical solution, we sought to surface shared challenges and opportunities across disciplines.

To this end, the workshop incorporated two participatory discussion sessions. (N = 16) participants from diverse disciplines engaged in semi-guided discussions responding to the following research questions:

- RQ1: Why has online violence against women not yet been effectively addressed?
- RQ2: What future directions and strategies are needed to tackle this problem?

Following the sessions, we applied thematic analysis [27] to identify key themes and priorities. Two dominant themes emerged. First, participants highlighted *accountability gaps* in how online platforms and governments respond to technology-facilitated gender-based violence, emphasising the concentration of power, misaligned economic incentives, weak enforcement, and regulatory lag that collectively enable the persistence of harm. Second, discussions underscored the *social and cultural dimensions* of the problem, particularly the difficulty of engaging allies and bystanders, especially men and boys, and the role of digital systems, educational practices, and social norms in normalising misogyny and limiting effective intervention. Together, these themes illustrate that addressing OVAW requires coordinated socio-technical responses that extend beyond platform-level fixes to encompass governance, design, and cultural change. Importantly, the discussions also generated a set of recommendations that synthesise multidisciplinary perspectives into priority actions, forming a key contribution of this work.

Thus, the main contributions of this article are the following:

- *Cross-disciplinary synthesis of the literature:* We provide a structured review across social, legal, and technical scholarship on OVAW, identifying tendencies and recurring conceptual gaps, such as clarifying the distinction between illegal harms and “harmful but not illegal” practices as central to understanding regulatory and accountability limitations.
- *Empirical interdisciplinary insights:* Drawing on participatory discussions from an interdisciplinary workshop with researchers and practitioners, we identify two central challenges that help explain the persistence of OVAW: (i) structural accountability gaps in platform governance and public regulation, and (ii) socio-technical dynamics that normalise misogyny and hinder effective bystander intervention.
- *Cross-disciplinary recommendations for action:* We synthesise the workshop outcomes into a set of recommendations spanning platform governance, regulatory design, algorithmic systems, and educational interventions, highlighting areas of convergence across disciplines that rarely engage collectively in addressing OVAW.

The remainder of this article is structured as follows. Section 2 reviews existing literature on OVAW. Section 3 details the workshop design and analysis methodology. Section 4 presents and discusses the key findings. Section 5 outlines key recommendations, and Sect. 6 concludes the work.

## Literature review

In this section, we present a literature review that explores some of the key challenges related to women’s online safety across multiple disciplines, including social, legal, and technological perspectives, which broadly reflect both the main strands of scholarship on OVAW and the disciplinary backgrounds represented among workshop participants. This review is not intended to be systematic; rather, it aims to capture prevailing tendencies and recurring challenges identified within these fields.

### Social

From a social science perspective, scholars point to two main challenges that contributes to the persistence of OVAW: (i) the digital reproduction of patriarchy in online spaces, and (ii) the multi-dimensional nature of violence against women.

*Digital reproduction of patriarchy in online spaces:* Feminist scholarship implicates the patriarchy and its reproduction, extended online as one of the core problems. Whether as a result of backlash to feminist progress [91] or as a new tool of misogyny [58], the feminist perspective grounds the ongoing violence against women, both on- and offline, as a product of patriarchy still evident across the globe. Norm-critical perspectives analyse phenomena through the lens of hegemonic norms and principles, often focusing on specific dimensions of a problem, such as masculinity and how masculinities develop across different times and contexts. For example, research may focus on the impacts of male fragility when faced with women’s liberation or empowerment [84], or the challenge of men’s identity formation in a changing world [63]. Zooming in on the individual and their environment, social learning theorists would suggest that we replicate what we see and learn from our environments [87]. This process is more than imitation. Rather, it occurs within specific social contexts where patriarchal norms are transmitted and reinforced. The online sphere represents a unique environment where these social learning dynamics play out, as individuals absorb and reproduce harmful behaviours they observe in both physical and digital spaces, particularly when these behaviours are consistent with broader patriarchal ideologies.

*Multi-dimensional nature of violence against women:* Multiple perspectives offer important lenses on the problem of continued violence against women, but none tell a complete story. Violence against women, on- and offline, is complicated by differences across regions in language [38], and definitions of harm and violence [89] that are influenced by cultural norms [95]. Add to this the challenge of technology that crosses borders and proliferates harmful content faster than it is resolved [23], and the many different legal frameworks that exist nationally and regionally. An Ecological Model from Bronfenbrenner [15] aggregates many of these perspectives into the idea that violence is a result of multiple interacting factors at different levels (the individual, their relationships, their communities and society). Individual factors may have to do with personal history (such as experiences of abuse or substance abuse). Relationship factors may include power asymmetries or other types of dependencies. Community factors may include an assessment of what kinds of resources are available, the extent of social isolation or alienation, and societal factors such as societal norms and specific legal environments. This approach highlights the complexity of violence and the need for multi-level interventions.

### Legal

On the legal side, the rise in OVAW [8, 52] has naturally prompted questions about, and calls for, legal regulation of online spaces. However, two key challenges can be highlighted in this space: (i) many harmful practices are not legally recognised and (ii) where regulation does exist, it often lacks enforceability.

*Regulation lacks enforceability:* In the past few years, specifically, we have observed the creation of online safety ‘hubs’ with bespoke legislation focusing on ‘online safety’ more generally, albeit inclusive of some forms of technology-facilitated violence against women. In the UK, the Online Safety Act 2023 [96] has been referred to as world-leading in its attempt to “make the UK the safest place in the world to be online” [14], following enactment of a comprehensive Online Safety Act 2021 in Australia [72] and Online Safety and Media Regulation (OSMR) Act 2022 in Ireland [44].

However, the creation of these online safety ‘hubs’ has resulted in only partial regulation of technology-facilitated violence against women, particularly where platform duties and accountability are concerned. For example, the UK’s Online Safety Act, whilst introducing new laws in some areas such as cyberflashing (making it a criminal offence) and stalking (making it a priority offence and therefore placing a duty on platforms to take proactive steps to tackle such content rather than only take it down), has underdelivered on its promise to advance women’s and girls’ online safety. As noted by leading organisations [21], there is a need for a greater and more specific focus on technology-facilitated violence against women within the Online Safety Act ecosystem, also in relation to codes of practice regarding child safety and illegal harms. A significant weakness lies in the Online Safety Act’s approach towards harmful content disproportionately affecting women and girls. Under the Online Safety Act, Ofcom^5^ is required to produce such guidance setting out how providers can take action against harmful content and activity and has already consulted on this matter [68]. However, whilst guidance is a welcome step, its key weakness lies in the lack of enforceability as a legally non-binding instrument. This approach marginalises women’s and girls’ online safety and places it in a non-governable realm by relying on good will of platform providers to follow such guidance with no implications for not doing so.

*Harmful practices not legally recognised:* A second key challenge is that not all harmful online content and acts that disproportionately affect women and girls have been made illegal, therefore, creating a category of “harmful but not illegal” and limiting avenues of redress for victims where the technology-facilitated violence they experienced falls into this category. Unless a form of technology-facilitated violence is named and enshrined within the law, it cannot be addressed by the law and its institutions, including the provision of legal remedies. This leads to the creation of a ‘hierarchy of harms’ whereby some forms of technology-facilitated violence against women and girls are privileged by law and actionable within it, whereas others, whilst harmful, are not, leaving victims without any recourse to legal remedies [7]. In this context, the hierarchy refers to the differential legal recognition of harms, whereby certain forms of abuse receive legislative attention and enforcement, while others remain marginalised within legal frameworks. As a result, harms such as text-based sexual abuse, threats, and gendered harassment (despite their documented psychological, reputational, and participatory impacts [7]) are often treated as less serious or insufficiently captured by existing legal provisions.

In addition, recently some of these regulations are being controversial, such as the US Allow States and Victims to Fight Online Sex Trafficking Act (FOSTA-SESTA)–legislation intended to combat online sex trafficking by increasing platform liability. Digital rights organisations, alongside sex worker-led research [42], argue that this law has driven sex work further underground, stripped workers of vital digital safety and screening tools, and ultimately reduced their safety. Paradoxically, these measures have also been shown to hinder legitimate law enforcement investigations by removing the online evidence previously used to track traffickers, thereby shifting harmful activities to less visible or overseas digital spaces.

These limitations reflect a global pattern of fragmented and uneven legal responses. Internationally, a significant number of countries still lack specific statutory provisions or dedicated regulatory frameworks that explicitly address technology-facilitated violence against women and girls [9].

### Technical

From a technological point of view, two key challenges have been identified: (i) the technological amplification of harms and misaligned incentives that design and optimise technology around profitability, and (ii) existing limitations of automated detection and mitigation systems.

*Technological amplification of harms and misaligned incentives:* Digital platforms and online systems are frequently designed and optimised around engagement-driven or performance-based metrics that prioritise profitability and efficiency, such as clicks, watch time, conversion rates, or predictive accuracy. A growing body of research shows that these optimisation objectives can unintentionally amplify misogynistic, polarising, or abusive content [4, 81], as such material often generates strong emotional responses and sustained user engagement. Although a growing body of research links engagement metrics to the spread of harmful content [4, 81], the extent to which outrage systematically drives amplification remains debated. Evidence from platform-scale audits and cross-platform analyses shows heterogeneous effects: amplification and user engagement varies with platform architecture, recommender design and users privacy [80, 106].

Beyond social media, algorithmic systems used in domains such as banking, recruitment, and credit scoring are commonly trained on historical data that reflect existing social biases [12, 28], resulting in outcomes that may reproduce or exacerbate gendered inequalities when fairness and safety are treated as secondary design considerations [12, 47, 57]. In addition to these dynamics, there is also the intentional and malicious misuse of digital technologies to perpetrate gender-based harm, including the use of generative image and video tools to produce non-consensual sexual content or abusive material [107]. While some forms of misuse are difficult to anticipate, others could have been mitigated through anticipatory risk assessment and safety-by-design measures. The rapid deployment of new technologies, often driven by competitive market pressures, can therefore outpace meaningful consideration of social harms, underscoring the need for more robust risk management, slower and more reflective development processes, and the systematic integration of gender-aware safety considerations into technological design, development and deployment.

*Limitations of automated detection and mitigation systems:* While multiple approaches have been developed to detect hate speech [109], harassment [93], and abusive behaviour [37], existing systems struggle to capture the contextual, implicit, and intersectional nature of technology-facilitated gender-based violence [56]. Harm may be expressed through coded language, humour, irony, or culturally specific references, making it difficult to identify using automated approaches alone [30, 36]. As a result, such systems often rely on simplified linguistic or behavioural signals that fail to reflect the complexity of gendered harm and may overlook abuse that does not conform to explicit or previously observed patterns.

In parallel, most algorithmic bias detection and mitigation mechanisms remain limited in their ability to address structural and intersectional forms of discrimination. Many fairness-aware approaches focus on narrow, formal definitions of bias or on protected attributes in isolation, overlooking how harms emerge at the intersection of gender with race, class, sexuality, or disability [103]. Bias audits are frequently conducted post hoc, rely on incomplete or proxy demographic data [3], and are rarely sustained over time as systems evolve. Moreover, mitigation techniques often do not address upstream design choices, data generation processes, or downstream social consequences [62, 66]. These limitations undermine the effectiveness of bias mitigation efforts and risk creating a false sense of safety. Systems may be perceived as “fair” or “neutral” simply because they satisfy a specific mathematical constraint, even while continuing to produce gendered and unequal outcomes [55]. This veneer of technical neutrality is particularly problematic in the context of OVAW, as it allows platforms to point to successful bias audits as evidence of safety while the underlying system architecture continues to facilitate abuse.

Finally, the governance and control mechanisms surrounding emerging technologies frequently lag behind their deployment. Many systems lack built-in safeguards to prevent foreseeable misuse, such as the generation of non-consensual sexual imagery [107]. The absence of robust auditing, monitoring, and redress mechanisms further limits the capacity to identify and address harms once they occur.

## Methodology

As part of the *TSWW'25* workshop, we conducted two structured discussion sessions designed to examine both the underlying reasons for the persistence and escalation of OVAW and to collectively identify potential pathways for addressing these challenges. A participatory approach was adopted to elicit knowledge from experts working across disciplinary and sectoral contexts, and to enable collective sense-making around shared challenges and priorities. We applied an adaptation of Participatory Action Research (PAR) from Chevalier and Buckles [24], to make the most out of the multi-sectoral, interdisciplinary participant profile of the conference. PAR grounds research in matters of social justice, involving the integration of life in society (participation), lived experience (Action) and knowledge creation (Research) to make advancement toward a shared problem, in our case, OVAW. Rather than dehumanising science, PAR “reconnects the creation of knowledge with the proverbial ‘human element’ [24]. Given the relatively short time with participants and the location of the workshop at an international conference, we applied a pragmatic approach to PAR that involves the facilitation of group exercises on problem assessment (including system dynamics and other types of analysis), logical sorting and priority setting.

*Participants:* Sixteen participants (N = 16) took part in the workshop. Participants were recruited through voluntary participation. The workshop was advertised in the conference program as an open session, and participants were conference attendees who voluntarily joined based on their professional interest in online safety. Participants included (N = 8) with backgrounds in computing, (N = 7) from the social sciences, and (N = 3) specialising in legal and governance domains, with several individuals spanning multiple disciplines. In addition to disciplinary diversity, the group reflected a range of professional sectors, including academia and education (N = 12), industry (N = 2), government (N = 1), and consultancy (N = 1). This combination of disciplinary and sectoral perspectives was key to the study. Bringing together participants who engage with these issues from different points of view enabled the examination of how responsibilities, constraints, and priorities are understood and contested. This diversity also helped surface tensions and complementarities that would be less visible within single-discipline studies.

*Session Design and Data Generation:* The one-day workshop centred around two research questions designed to structure the participatory discussions and facilitate the exchange of perspectives across disciplines. The research questions were developed during the workshop design phase through iterative discussions among the organisers, a multidisciplinary group of researchers and practitioners specialising in online violence, digital governance, and responsible AI. The questions were refined to encourage open and cross-disciplinary discussion on the challenges and future directions for addressing OVAW.^6^

The first session centred on the question: “Why has online violence against women not yet been effectively addressed?” To initiate discussion, participants engaged in a structured brainstorming exercise in which they individually identified what they perceived to be the most pressing challenges in addressing online gender-based violence.

Contributions were openly shared and documented on a collaborative whiteboard, creating a shared artefact that captured participants’ framing of the problem space. This clustering process was conducted transparently and iteratively, with participants able to question, refine, or reassign items. Participants were then asked to collectively prioritise the resulting clusters according to perceived importance and urgency. This process yielded two overarching thematic areas, ordered by participant prioritisation:

1. *Platforms and Government:* It refers to the ongoing failure of online platforms to effectively moderate harmful content, compounded by inadequate regulation and weak enforcement mechanisms from governments. This theme also included issues of accountability, transparency, and lack of coordination between regulatory bodies and tech companies.
2. *Socio-Technical Drivers of Online Misogyny and Barriers to Ally Engagement:* It refers to the socio-technical mechanisms that enable the production, normalisation, and persistence of online misogyny, alongside the challenges of engaging allies and bystanders in response.

**Fig. 1:**
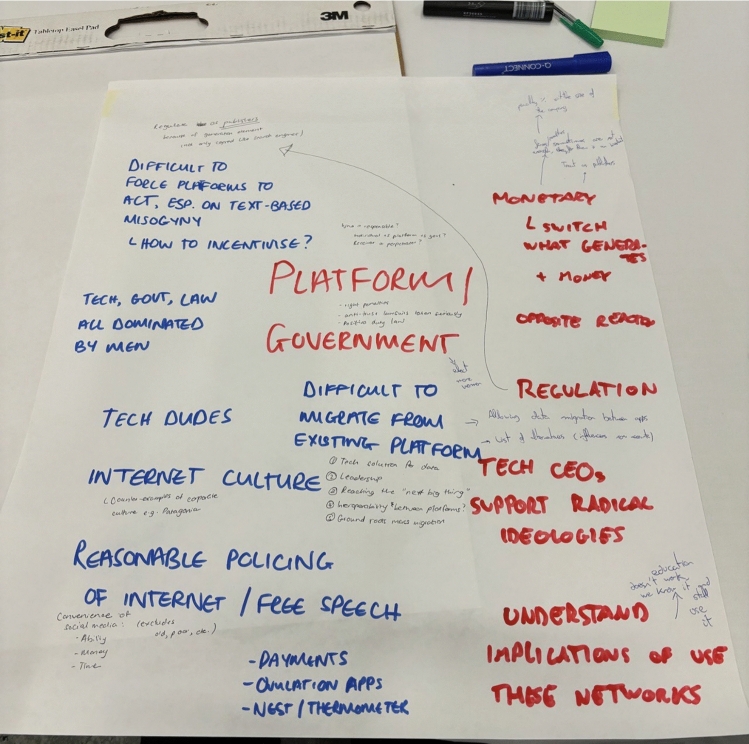
Collaborative whiteboard from *TSWW'25* workshop, where participants identified key challenges in tackling OVAW

Once challenges were articulated, the session transitioned into a facilitated large-group discussion structured around these two themes and two workshop moderators (both co-authors of this paper) facilitated an affinity-mapping exercise [86] on a collaborative whiteboard (Fig. 1), clustering contributions based on shared underlying issues. Participants were invited to reflect on why these challenges persist despite existing initiatives and policy efforts. Moderators encouraged contributions from across disciplinary perspectives, and detailed notes were taken to capture both convergent and divergent viewpoints.

The second session addressed the complementary question: *“What future directions and strategies are needed to tackle this problem?"* Building on the challenges identified in the first session, participants were asked to propose concrete actions, structural changes, or research and policy priorities that could meaningfully address the issues discussed. Contributions were again generated through open discussion and documented collaboratively through an affinity-mapping exercise [86] on the collaborative whiteboard. This session was explicitly oriented toward identifying actionable and forward-looking recommendations.

*Data Characteristics and Ethical Considerations:* Data for this study consist of collaboratively produced workshop artefacts (including the collaborative whiteboard) and detailed facilitator notes taken during both sessions, with participants’ informed consent. We followed the Lead Facilitator-Scribe model [53]: one co-author acted as a dedicated scribe throughout the workshop, while the other two facilitators focused on guiding the discussion. The scribe’s note-taking followed a structured framework adapted from Schatzman and Strauss, prioritising observational notes to systematically record participant dialogue [85]. To enhance the depth of the collected data, these notes were expanded by the facilitators into full reflective field notes [33] within 24 h of the workshop’s conclusion.

Data were note-based rather than audio-recorded or transcribed verbatim. This methodological decision was taken deliberately in light of the sensitivity of the topic and the likelihood that participants would draw on professional or personal vulnerable experiences. While this approach limits access to fine-grained interactional detail (e.g. tone or the texture of disagreement), it prioritises participant comfort and is consistent with established qualitative workshop methodologies [73]. Accordingly, the analysis emphasises thematic patterns rather than detailed discourse dynamics. To enhance analytic rigour, three authors independently coded the data before engaging in iterative comparison and discussion. Disagreements in coding or theme interpretation were resolved through deliberation until consensus was reached. To support analytic transparency, we include exemplar extracts from the collaboratively generated artefacts (see Fig. 1), which directly reflect participants’ own language and conceptualisations.

*Data Analysis:* To analyse the data collected from both sessions, we applied thematic analysis, following the six-phase approach developed by Clarke and Braun [27]. This method allows for the identification and interpretation of patterns and themes within qualitative data. The analysis was conducted by three authors of this paper and proceeded as follows:

1. *Familiarisation:* Three coders independently reviewed the raw data from both sessions, including written notes and whiteboard content.
2. *Initial coding:* Each coder conducted inductive, open coding on each contribution, assigning short phrases or tags to capture key ideas (e.g., “lack of enforcement,” “ally fatigue,” “algorithmic opacity”).
3. *Theme generation:* The three coders then compared their initial code sets, discussing areas of overlap, divergence, and interpretation. Through iterative discussion, codes were refined, merged, or clarified. Using the consolidated codes, the coders collaboratively grouped related codes into candidate themes that captured recurring patterns across the dataset.
4. *Reviewing themes:* Themes were refined and consolidated to ensure internal consistency and distinction between them. Some themes were merged, while others were split into subthemes for clarity.
5. *Defining and naming themes:* Each theme was given a clear, descriptive label and a definition grounded in the data.
6. *Producing the report:* The final themes were used to structure the analysis presented in the next section.

Throughout the analysis, discrepancies in interpretation were discussed among the authors until agreement was reached.

*Situating the Findings:* This approach allowed us to analyse the interdisciplinary knowledge produced during the workshop and to translate collective insights into a structured understanding of both challenges and opportunities in tackling OVAW. Relevant academic literature and policy documents were consulted to contextualise and interpret the workshop findings. These sources were used to triangulate participants’ insights and situate the findings within broader scholarly and policy debates. They were not treated as primary data but as interpretive resources. The findings of our analysis are discussed in the next section.

## Findings

In this section, we present and reflect on the main findings that emerged from the discussions. Specifically, we examine the challenges that continue to hinder meaningful progress in tackling OVAW, as identified and prioritised by participants. We also explore the future directions and strategies proposed by attendees for addressing these persistent barriers.

Many of the challenges raised by participants reflect broader, structural issues highlighted in feminist scholarship on violence against women. Policy responses have increasingly shifted toward individualised criminal justice approaches that treat violence primarily as the actions of individual offenders rather than as a product of broader structural inequalities [46]. This shift can obscure systemic drivers and limit the effectiveness of interventions. Research also indicates that many successful responses to violence occur outside formal legal systems, including community-based initiatives and long-term survivor support, while emphasising the need to address intersecting inequalities related to gender, race, and class [46]. These insights resonate with the workshop discussions, where participants emphasised the structural and socio-technical dimensions of OVAW.

To organise these insights, the discussion is structured around the two main thematic areas derived during the workshop: (i) Platforms and Governments and (ii) Socio-Technical Drivers of Online Misogyny and Barriers to Ally Engagement. Each subsection outlines the nature of the challenge, explores the underlying dynamics that sustain it, and offers suggestions for mitigation or transformation.

### Platforms and governments: accountability, power, and structural inertia

The most prominent theme raised during the workshop was the role of tech companies and governments in perpetuating, neglecting, or failing to respond adequately to OVAW. At the centre of this discussion was the question of who bears responsibility for preventing and addressing such harms? individual users, platform providers, state institutions... The consensus was that responsibility cannot be individualised alone; it must be structurally distributed, with platforms and governments playing decisive roles in both prevention and accountability.

#### Profit over protection

Participants pointed out that many platforms have economic incentives to allow the continued presence of radicalised and harmful content. Content that generates strong emotional responses (e.g. content that promotes misogyny, outrage, or polarisation) tends to increase user engagement [64], which in turn boosts advertising revenue. One frequently cited example was the widespread monetisation of misogynistic influencers like Andrew Tate, whose content remains easily accessible and profitable for platforms despite public outcry [94]. In such cases, monetary fines to platforms for harmful content often serve as mere costs of doing business rather than effective deterrents. As a solution, participants pointed out that penalties for negligence or harm should be proportional to the size and revenue of the company, for example, calculated as a percentage of annual turnover. For example, under the UK Online Safety Act 2023 [96] and the EU AI Act 2024 [99], companies can face fines of up to $$\pounds $$18 million or 10% of their worldwide annual turnover, and up to €35 million or 7% of turnover, respectively. However, some jurisdictions still rely on fixed-amount penalties that ignore company revenue. For instance, Germany’s Network Enforcement Act (NetzDG) sets a cap of €50 million [17], and Australia’s Online Safety Act 2021 imposes a maximum of 500 penalty units (about A$782,500) [72].

Additionally, other participants raised the idea of reclassifying platforms as publishers. Unlike search engines, which merely index external content, social platforms host, amplify, and algorithmically curate user content. This active role implies editorial responsibility, comparable to traditional media outlets. By regulating platforms as publishers, governments could impose stricter liability for the dissemination of harmful or illegal content, similar to how copyright enforcement mechanisms currently function.

#### A culture of resistance and power imbalances

Participants also expressed deep concern about the lack of willingness from powerful stakeholders in the tech industry to meaningfully engage with these issues. For example, the leadership of major platforms, including X (formerly Twitter), has in some cases openly supported actions and ideologies that are dismissive, or even hostile toward anti-misogyny efforts [39, 82]. This resistance is exacerbated by the homogeneity of those in leadership and development roles, who are often men unaware of the lived experiences of online harassment or its gendered dimensions [71]. A similar imbalance exists in government and policymaking circles, where women remain globally underrepresented in key decision-making positions [50].

Furthermore, participants mentioned that many technology companies state commitments to ethics, safety, and inclusion, but these commitments have often proven to be fragile once priorities or leadership change. X (formerly known as Twitter) dismantled the independent trust-and-safety council after a leadership change [29]. Google has also changed their AI principles, allowing concerning exceptions that weaken its original ethical commitments to responsible development and use of AI [49]. Meta disbanded its responsible innovation group tasked with spotting product harms [60]. And Microsoft eliminated the ethics team meant to ensure its own Responsible AI principles were implemented in practice [104]. Together, these reversals show that inclusion isn’t a durable value if it can be revoked whenever it conflicts with profit, politics or speed-to-market.

Scholars have identified several structural and institutional reasons for this consistent pattern of dismantling ethical infrastructures. First, ethics teams in the tech industry are often ’decoupled’ from the primary product teams, meaning they lack the institutional authority to stop or meaningfully pivot a product launch that violates safety standards. This structural isolation is driven by a market that prioritises speed-to-market and high-engagement metrics above all else; when ethical recommendations threaten to delay a launch or reduce engagement, they are viewed as ’productivity friction’ rather than a core business requirement [2]. Secondly, the use of ethics to satisfy public relations or regulatory pressure means that these infrastructures are treated as static compliance exercises rather than integrated socio-technical commitments. Consequently, when leadership shifts or economic pressures mount, these ’auxiliary’ teams are the first to be dismantled as they are perceived as non-essential to the company’s survival in a hyper-competitive market [2, 10].

#### Freedom of speech vs. policing online harm

Participants discussed the ongoing challenge of how to reduce online harms while still protecting freedom of expression. They highlighted that strict or poorly designed speech regulations can have unintended consequences, especially for marginalised groups. Actually, research has shown that content moderation systems often disproportionately remove the posts of racialised women, even when these posts do not violate platform rules [45]. Black and transgender users, for example, frequently report that their content about racism or queer identity is taken down despite following guidelines [45]. These examples illustrate how well-meaning regulations can silence those most in need of protection, and suggest that any effort to moderate speech must be carefully designed to avoid reinforcing structural inequalities.

At the same time, participants strongly argued that protecting women from online abuse should not be treated as a threat to freedom of speech. Instead, safety should be seen as a necessary condition for free expression. Gender-based abuse online (e.g. harassment, doxxing, or online threats) has been widely documented as a barrier to participation in digital life [26, 78]. Many victims, particularly women, are pushed out of public conversations because of repeated targeting. Addressing this kind of harm is not about limiting speech, but about making it possible for more people to speak freely in the first place.

These dynamics demonstrate that freedom of expression and protection from harm are not competing principles but mutually dependent conditions within socio-technical systems. The challenge, therefore, lies in designing moderation infrastructures and regulatory regimes that reduce gendered abuse without reproducing existing structural inequalities.

#### Inertia and entrenchment of established platforms

A practical barrier raised was the difficulty of user migration away from harmful platforms. Once users are embedded in a platform (e.g. with social ties, content history, or audience reach) it becomes challenging to leave, even when the environment becomes hostile [65]. As a result, more ethical or safer alternatives often struggle to gain traction. Participants proposed several solutions:

- Tech-enabled data portability and interoperability, which would allow users to transfer their social graphs and content across platforms, provided that such transfers respect privacy regulations and protect the rights of individuals whose data may be included in those social connections.
- Policy interventions requiring platforms to facilitate such transfers (analogous to open banking or mobile number portability).
- Grassroots migration efforts, such as the Twitter–Bluesky shift [13], and creator-led initiatives, where content creators share profiles across platforms to support community transitions.

#### Broader tech accountability

The group stressed that harmful dynamics are not limited to online platforms, such as social media. Other technologies, such as banking apps or ovulation trackers, can also be used to exert control over women; financially, medically, or emotionally. In one example, perpetrators have exploited banking applications to monitor or restrict a victim’s financial activities [67, 88]. Digital fertility tracking apps have also raised concerns due to the sensitive personal data they collect and share online, which could be exploited for surveillance and control [19, 59]. Even smart home technologies have reportedly been misused by abusers to remotely manipulate the home environment and gaslight victims (e.g., by increasing and decreasing the home temperature, or the temperature of water, making victims think they are going crazy) [75]. This phenomenon underscores the ’gender-blind’ nature of early IoT development. By designing smart systems under the assumption of a ’harmonious household,’ developers failed to account for adversarial use by a perpetrator. When the user is the perpetrator and the ’subject’ is the victim, the system’s efficiency (its ability to change temperatures instantly and silently from a distance) becomes a harmful feature. These cases point to a broader need for gender-aware design, deployment and monitoring practices in all areas of tech development, particularly where personal or biometric data is involved.

#### Enforcement and incentivisation

As discussed in the literature (see Sect. 2), participants also highlighted that enforcement remains one of the weakest links in efforts to combat online misogyny, particularly text-based harm, which is less visually explicit but often more pervasive and damaging over time. For example, multiple studies and regulators document persistent under-enforcement against misogynistic harassment and “pile-ons” (the action of followers who join a hostile group in harshly criticising or judging a less dominant group or an individual), contributing to significant mental-health effects [52, 69, 78]. Participants advocated for stronger enforcement through:

- Proportional financial penalties (e.g., fines as a percentage of revenue). For example, the UK Online Safety Act empowers Ofcom to fine services up to £18 million or 10% of global revenue [96].
- Positive duty laws, requiring platforms to actively prevent harm rather than simply react to it. For example, the UK Online Safety Act introduces systemic duties of care and Ofcom’s guidance sets proactive expectations to prevent misogynistic “pile-ons” [69].
- Anti-trust litigation or regulatory action against platform monopolies that abuse their dominance. When a small number of powerful companies control the digital ecosystem, they can shape the rules of moderation. Competition enforcement can therefore be a tool not just to constrain economic dominance, but to unlock better enforcement outcomes by creating more accountable and diverse digital environments.

#### The law moves slowly (but it doesn’t have to)

Another major concern was the inadequacy and slowness of legal frameworks, especially in addressing new technological threats. The regulation of deepfakes was cited as a prime example: despite widespread awareness of their misuse to create non-consensual sexual content, most jurisdictions still lack targeted legal frameworks [79, 102] to address them, or have only introduced such measures long after the technology became widely accessible and harmful [43, 105].

Participants stressed the importance of interdisciplinary foresight and early collaboration to anticipate risks and help shape legislation proactively. This would counteract the current tendency for law to follow technological development rather than guide it.

### Socio-technical drivers of online misogyny and barriers to ally engagement

The second key theme emerging from the workshop concerned the socio-technical processes through which online misogyny is produced, normalised, and sustained, and the resulting challenges of mobilising allies and bystanders. Participants emphasised that online gender-based violence cannot be understood solely as a problem of individual behaviour or victimisation, but rather as the outcome of interacting social, algorithmic, and cultural mechanisms. These include the silencing of women’s voices in digital public spaces, the algorithmic amplification of misogynistic content, early socialisation into harmful norms, and structural tendencies to shift responsibility for safety onto those most affected. Within this context, men and boys frequently remain disengaged as bystanders or are actively drawn into harmful online cultures. Participants therefore stressed that meaningful change depends on interventions that reshape social norms, redesign digital systems, and introduce early educational and socialisation strategies that promote empathy, accountability, and critical engagement.

#### Gendered visibility and the silencing of women online

Participants emphasised that women voices are increasingly being silenced online, with digital spaces becoming more hostile and polarised [40, 92]. Platforms such as X (formerly Twitter) were cited as examples where public discourse has grown more conservative and less tolerant of feminist voices. The case of women politicians was raised to illustrate how misogynistic abuse disproportionately targets women in public-facing roles, further deterring women participation and visibility in online political discourse [41, 83, 90]. Participants also expressed concern over the backlash effect, in which increased advocacy for gender equality online paradoxically results in intensified hate and resistance [5, 31].

#### Algorithmic amplification and the production of harmful masculinities

Participants mentioned that teenagers and young men often see online personalities as role models, so those who dominate their feeds play a key role in shaping ideas about masculinity. On social media platforms, algorithms rapidly promote and reinforce manosphere content to male users. In particular, new male accounts on platforms like YouTube and TikTok have been shown to receive misogynistic and extremist content within as little as 20 min [4, 81]. This finding emerged from systematic testing where researchers monitored the algorithmic output of new created profiles to evaluate the default recommendations provided to young male users, demonstrating that even without a long-term browsing history, the algorithmic default for young men leans toward ’manosphere’ content. Although other influencers promote gender equality and healthier forms of masculinity, they are not commonly recommended by platform algorithms in the same way, as they don’t generate the same engagement [4, 80], which ends up restricting their ability to compete with more harmful voices.

In targeting this problem, participants proposed several mitigation strategies. Metrics used to evaluate algorithmic systems behind the platforms, particularly recommender systems, need to shift from engagement time and click-through rates to include considerations of user well-being, public safety, and mental health. Participants also suggested giving users more agency, such as robust and effective “do not recommend this” tools, and even more proactive mechanisms for managing content exposure. Algorithmic evaluation frameworks must balance commercial interests with ethical and social responsibility.

#### Vulnerability, belonging, and pathways into radicalisation

The group explored why young users are drawn to radicalised communities in the first place. A key insight was that social isolation and polarisation create fertile ground for such engagement. Isolated individuals, especially young men lacking support or belonging, are more vulnerable to online grooming by radical groups [76]. Participants noted that future research should not only trace the exposure to harmful content, but also map the pathways through which individuals become radicalised. By identifying the trigger points or unmet needs that drive users into these communities, interventions can be better tailored to disrupt the recruitment cycle.

#### Socialisation, education, and early intervention

Participants emphasised that educational interventions [1, 34, 70] must begin at an earlier age than they currently do. Discussions with young people about misogyny, online harm, and empathy are often introduced only after exposure has already occurred. By the age of 12, many children have spent several years navigating digital environments, during which harmful norms and behaviours may already have been normalised. As a result, participants advocated for age-appropriate guidance from the earliest stages of digital engagement, focusing not only on personal safety but also on critical engagement with online content and strategies for responding to harmful narratives when they arise. Media literacy and social–emotional learning from a young age were viewed as especially important for challenging damaging online norms and fostering safer digital environments for women and girls.

This emphasis on early education stood in contrast to the reliance on parental controls or blanket bans, which participants widely regarded as ineffective. Such measures were seen as easily circumvented, socially isolating, or insufficient in addressing the underlying attitudes and behaviours that enable online misogyny. Moreover, children and adolescents are frequently exposed to harmful content through peers, even when formal restrictions are in place. Participants therefore argued that restrictive approaches must be complemented, or in some cases replaced, by educational strategies that equip young people with the skills to navigate, question, and resist harmful content rather than simply attempting to shield them from it.

Beyond formal education, participants highlighted socialisation as a critical and often overlooked factor in shaping online behaviour. Norms around gender, masculinity, and acceptable conduct are learned and reinforced through everyday interactions with peers, family members, educators, and online communities. When misogynistic language or harassment is tolerated, minimised, or dismissed, it becomes embedded as a normal aspect of digital culture. Participants stressed the importance of engaging not only children but also parents, teachers, and youth workers in fostering norms of empathy, accountability, and active bystander intervention. In this view, preventing online gender-based violence requires sustained socialisation processes that challenge harmful norms across both online and offline contexts, rather than isolated educational interventions alone.

#### The persistent shifting of responsibility onto victims

The group discussed how victims, especially women and girls, are continuously pressured to adapt their online behaviour to avoid abuse. This includes self-censorship, limiting visibility, or refraining from posting certain types of content [52]. These dynamics are not new: they echo decades of victim-blaming discourses (e.g. “what were you wearing?” or “why were you out so late?") that normalise sexual violence and shift accountability from perpetrators to those targeted [18]. Such pressures reinforce harmful norms and effectively place the burden of safety on those most at risk, rather than addressing the root causes of harm. Instead, participants called for a “protection by design” approach, in which digital environments are proactively structured to prioritise safety and inclusion, reducing the need for self-protective behaviour by those most vulnerable to abuse.

#### Normalisation of misogyny and the challenge of cultural change

A final concern raised by participants was the normalisation of hate speech, particularly misogyny, within online environments. Many observed that gender-based abuse has become so pervasive that it is frequently treated as an unavoidable feature of digital spaces rather than as a systemic problem requiring intervention [52]. Feminist scholarship similarly highlights how misogyny has become normalised within digital cultures, often framed as humour or trolling, this abuse is minimised, which discourages reporting and intervention and contributes to women being pushed out of online public spaces [51]. This normalisation contributes to widespread desensitisation, in which repeated exposure to misogynistic language and harassment reduces perceptions of harm and lowers expectations of accountability. As a result, abusive behaviour is often minimised, dismissed as “part of the internet,” or reframed as acceptable humour, opinion, or free expression.

Participants noted that such normalisation not only harms those directly targeted but also shapes bystander behaviour, discouraging intervention and reinforcing passive complicity. When misogyny is perceived as inevitable, reporting is seen as futile and resistance as socially costly. These dynamics disproportionately affect women and marginalised groups, who may withdraw from online spaces altogether, thereby narrowing the diversity of voices in digital public discourse. Participants stressed that addressing this challenge requires more than content moderation or individual sanctions; it demands sustained cultural change that challenges the underlying social norms enabling gender-based abuse.

In this context, participants highlighted the importance of long-term strategies aimed at reshaping expectations of acceptable behaviour online. Media literacy and public discourse that explicitly name misogyny as harmful were viewed as essential tools for countering normalisation and fostering collective responsibility.

## Key recommendations

Across the workshop discussions, participants collectively articulated a set of fifteen structural recommendations aimed at addressing the persistent failure to prevent and respond to online gender-based violence. While the foundational ideas and priorities were identified and debated by participants during the sessions, the final set of fifteen recommendations was synthesised by the authors during the thematic analysis of the workshop data. These recommendations are not presented as an exhaustive or definitive solution set; rather, they emerged from dialogue among participants with diverse disciplinary, professional, and lived-experience expertise. Their significance lies precisely in this grounding. The recommendations reflect points of convergence across distinct perspectives and thus capture priorities that cut across disciplines. In positioning these recommendations as the primary outcome of the workshop, we argue that their value rests not in their novelty in isolation, but in their collective articulation.

1. *Reform platform accountability frameworks:* Participants emphasised the need to move beyond voluntary self-regulation toward enforceable accountability mechanisms. This included proportional financial penalties tied to company revenue, positive duties of care requiring platforms to prevent harm proactively, and, where appropriate, the reclassification of platforms as publishers with corresponding liability for amplified content.
2. *Align economic incentives with user safety:* A recurring concern was that current platform business models reward engagement-driven amplification of misogynistic and polarising content. Participants argued that regulatory and enforcement mechanisms must directly counteract these incentives, ensuring that user safety and harm prevention are not systematically deprioritised in favour of profit.
3. *Strengthen enforcement and reduce regulatory fragmentation:* Participants highlighted enforcement as a primary weakness. They called for better-resourced regulators, clearer enforcement mandates, and stronger international coordination to address the cross-border nature of online harms.
4. *Adopt safety-by-design and gender-aware approaches across technologies:* Responsibility should extend beyond social media platforms to encompass the broader digital ecosystem, including financial technologies, health and fertility apps, and smart home systems. Participants stressed the need for gender-aware design, risk assessment, and monitoring practices.
5. *Enable meaningful platform exit through interoperability and portability:* To counter platform lock-in, participants advocated for privacy-preserving data portability and interoperability, supported by policy interventions that require dominant platforms to facilitate user migration.
6. *Reframe freedom of expression debates around inclusion and participation:* Rather than positioning safety and free speech as opposing values, participants argued that protection from gender-based abuse is a prerequisite for meaningful participation in digital public life, particularly for women and marginalised groups.
7. *Embed anticipatory and interdisciplinary governance practices:* Participants called for earlier and deeper collaboration between technologists, legal scholars, gender experts, and policymakers to anticipate emerging harms before they become widespread, rather than relying on reactive regulatory responses.
8. *Centre diverse women’s lived experiences:* Participants emphasised that increasing women’s representation in digital decision-making is necessary but insufficient without accounting for the diversity of women’s lived experiences. Participants therefore called for participatory governance and co-design processes that meaningfully involve women from marginalised and underrepresented groups throughout technology design, regulation, and enforcement. In practice, this could look like the institutionalisation of permanent advisory boards (similar to those used in governmental or intergovernmental bodies) that are consulted continually to identify ‘blind spots’ throughout the technology lifecycle. While achieving a representative balance within organisations is challenging, the integration of representative advisory boards could provide a mechanism to bring the voices of diverse groups of women directly into decision-making processes. These boards, which could be convened by national regulators and funded through mandatory industry levies, would ensure that diverse opinions are structurally embedded in design and policy rather than treated as a secondary or reactive consideration.
9. *Redesign algorithmic systems to reduce the amplification of harmful norms:* Participants stressed the need to move beyond engagement-driven optimisation in recommender systems, which currently amplifies misogynistic and polarising content. They called for evaluation frameworks that incorporate user well-being, public safety, and social impact, alongside mechanisms that give users greater agency over content exposure.
10. *Increase the visibility of pro-social and gender-equitable role models: * To counter the dominance of manosphere influencers, participants highlighted the importance of elevating alternative narratives around masculinity, empathy, and respect. This includes platform-level interventions that ensure content promoting gender equality and healthier masculinities is not disadvantaged by algorithmic systems.
11. *Shift responsibility away from victims through protection-by-design approaches:* Participants emphasised that safety should not depend on women’s self-censorship or withdrawal from online spaces. Instead, platforms should be designed to proactively reduce exposure to harassment and abuse, embedding protections that minimise the burden on those most frequently targeted.
12. *Invest in early, sustained digital citizenship and media literacy:* Participants called for age-appropriate, proactive interventions from the earliest stages of digital engagement. This focuses on equipping young people with the critical thinking skills to deconstruct harmful content and developing empathy-based digital socialisation. Practitioners (e.g., educators and parents) should prioritise media literacy and critical engagement over purely restrictive measures like bans or filters.
13. *Implement interventions to address social isolation and pathways into radicalisation:* Beyond content moderation, participants stressed the need to address the social and emotional drivers that lead young men toward extremist communities. Practitioners (e.g., youth workers and social services) should develop support systems based on research that target underlying drivers such as social isolation, lack of belonging, and identity formation.
14. *Reframe online misogyny as a collective and cultural problem:* Finally, participants argued that normalised misogyny must be challenged as a systemic issue rather than an inevitable feature of digital life. They emphasised the role of bystanders, allies, and institutions in disrupting passive complicity and fostering shared norms of accountability, dignity, and inclusion. Long-term cultural change was seen as essential for enabling meaningful participation in digital public spaces for women and marginalised groups.
15. *Foster sustained cross-sector and interdisciplinary collaboration:* Participants emphasised that OVAW is a fundamentally complex socio-technical problem that cannot be effectively addressed within disciplinary or sectoral silos. They called for sustained collaboration across academia, industry, civil society, and government, bringing together expertise from computer science, social sciences, law, education, and frontline support services. Such collaboration was seen as essential for aligning technical design choices with legal obligations, social realities, and lived experiences, and for ensuring that interventions are both context-sensitive and practically enforceable. Participants stressed that ad hoc consultations or short-term partnerships are insufficient; instead, long-term structures for interdisciplinary cooperation are needed to enable shared learning, coordinated responses, and the co-development of interventions that can adapt to evolving technologies, cultural contexts, and forms of harm.

The prioritisation of these recommendations is fundamentally context-dependent. We have intentionally avoided an explicit ranking to allow readers and stakeholders to determine which interventions offer the greatest capacity for impact within their specific regulatory, sectoral, and institutional environments. By presenting these priorities as a comprehensive but non-hierarchical set, we invite policymakers and practitioners to target those recommendations that best align with their specific context and their current capacity to resolve the systemic drivers of online violence.

## Conclusions

OVAW remains a pervasive and escalating global problem, shaped by the interaction of technological systems, governance, and deeply embedded social norms. While prior research has generated valuable insights within individual disciplines, efforts to address these forms of harm have often remained fragmented, limiting their practical impact. This paper contributes to the field by convening and systematically analysing perspectives from researchers and practitioners across technical, legal, and social domains–groups that rarely engage collectively or are required to articulate shared priorities. Although we did not ask about participants’ personal experiences, we recognise that important voices may not have been present in the workshop, including survivors of online abuse, and that representation from the Global South was limited (N = 1). Their absence necessarily shapes the contours of the consensus presented here and highlights the need for future work grounded more directly in these perspectives.

Through participatory discussion and thematic analysis, we not only identified persistent structural and cultural challenges, but also synthesised a set of actionable, cross-cutting recommendations that reflect areas of convergence across disciplines. These recommendations represent a key contribution of this work, translating diverse expertise into concrete directions for AI development, platform governance, and public policy. By demonstrating the value of interdisciplinary consensus-building, this study provides a foundation for more coordinated and effective responses to online gender-based violence.

## Data Availability

No datasets were generated or analysed during the current study.

## Footnotes

1 When we refer to women in this work, we use the term in a gender-inclusive sense, including cisgender and transgender women of all ages.

2 Definitions of OVAW differ across regions and studies, with variation in the behaviours included or the thresholds of harm recognised. In this study, we define OVAW as any act of gender-based violence against women that is committed, assisted or aggravated in part or fully by the use of ICT, such as mobile phones and smartphones, the Internet, social media platforms or email, against a woman because she is a woman, or which affects women disproportionately [101]. In this context, gender-based violence refers to harmful acts (e.g. sexual, physical, mental and economic harms) directed at an individual or a group of individuals based on their gender [100].

3 Technology-facilitated gender-based violence is ‘any act that is committed, assisted, aggravated or amplified by the use of ICTs or other digital tools, that results in or is likely to result in physical, sexual, psychological, social, political or economic harm, or other infringements of rights and freedoms [108]. TFGBV is a broader term than OVAW that includes the use of any type of technology (e.g. digital cameras, location tracking devices, etc.). OVAW primarily focuses on violence happening in digital spaces [108].

4 For the purposes of this paper, we conceptualise AI-based systems as a machine-based system that is designed to operate with varying levels of autonomy and that may exhibit adaptiveness after deployment, and that, for explicit or implicit objectives, infers, from the input it receives, how to generate outputs such as predictions, content, recommendations, or decisions that can influence physical or virtual environments [99].

5 Ofcom (Office of Communications) is the independent regulatory and competition authority for the UK’s communications industries. It regulates TV, radio, video-on-demand, fixed-line telecoms, mobiles, postal services, and, under the Online Safety Act, online platforms. http://www.ofcom.org.uk

6 For the purposes of the workshop, OVAW was defined according to the definition presented at the beginning of this paper, which was shared with participants at the start of the session.
